# Dysregulation of CD177^+^ neutrophils on intraepithelial lymphocytes exacerbates gut inflammation via decreasing microbiota-derived DMF

**DOI:** 10.1080/19490976.2023.2172668

**Published:** 2023-02-02

**Authors:** Huimin Chen, Xiaohan Wu, Ruicong Sun, Huiying Lu, Ritian Lin, Xiang Gao, Gengfeng Li, Zhongsheng Feng, Ruixin Zhu, Yao Yao, Baisui Feng, Zhanju Liu

**Affiliations:** a Center for Inflammatory Bowel Disease Research, The Shanghai Tenth People’s Hospital, Tongji University School of Medicine, Shanghai, China; b Department of Bioinformatics, School of Life Sciences and Technology, Tongji University, Shanghai, China; c Department of Gastroenterology, The Second Affiliated Hospital of Zhengzhou University, Zhengzhou, China; d Division of Immunology, School of Basic Medical Sciences, Henan University of Science and Technology, Luoyang, China

**Keywords:** Neutrophils, intraepithelial lymphocytes, CD177, dimethyl fumarate, intestinal inflammation

## Abstract

Neutrophils synergize with intestinal resident intraepithelial lymphocytes (IELs) to serve as the first-line defense and maintain intestinal homeostasis. However, the underlying mechanisms whereby neutrophils regulate IELs to inhibit intestinal inflammation are still not completely understood. Here, we found that depletion of neutrophils (especially CD177^+^ subset) caused expansion of colitogenic TCRγδ^+^CD8αα^+^ IELs, increased intestinal inflammation, and dysbiosis after dextran sulfate sodium exposure or *Citrobacter rodentium* infection in mice. scRNA-seq analysis revealed a pyroptosis-related gene signature and hyperresponsiveness to microbiota in TCRγδ^+^CD8αα^+^ IELs from colitic *Cd177**^−/−^*** mice. Microbiota-derived fumarate and its derivative dimethyl fumarate (DMF), as well as fumarate-producing microbiotas, decreased in the feces of colitic *Cd177**^−/−^*** mice. Elimination of dysbiosis by antibiotics treatment or co-housing procedure and DMF supplementation restrained TCRγδ^+^CD8αα^+^ IEL activation. Consistently, DMF significantly alleviated intestinal mucosal inflammation in mice through restricting gasdermin D (GSDMD)-induced pyroptosis of TCRγδ^+^CD8αα^+^ IELs. Therefore, our data reveal that neutrophils inhibit intestinal inflammation by promoting microbiota-derived DMF to regulate TCRγδ^+^CD8αα^+^ IEL activation in a GSDMD-mediated pyroptosis-dependent manner, and that DMF may serve as a therapeutic target for the management of intestinal inflammation.

## Graphical Abstract

**Figure:**
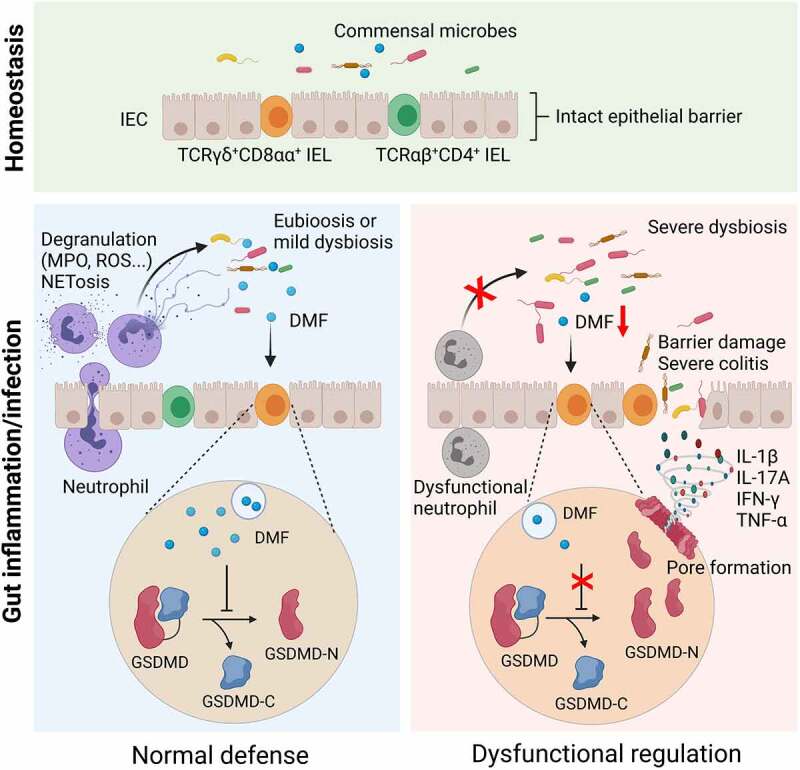

## Introduction

Neutrophils are the early responders to inflammatory signals and are crucial in immune regulation, pathogen clearance, and recruitment of immune cells in the gastrointestinal tract.^1,2^ Dysfunction of neutrophils contributes to impaired intestinal immune homeostasis and aggravated mucosal inflammation when pathogens invade. The underlying mechanisms involved in the dysregulated neutrophils include proinflammatory cytokine burst, irreducible immune responses, and discordant immune cell functions.^3^ CD177 (NB1 or PRV1) is a glycosylphosphatidylinositol-linked glycoprotein expressed exclusively in neutrophils, neutrophilic metamyelocytes, and myelocytes, which binds platelet endothelial cell adhesion molecule-1 and functions in modulating neutrophil transmigration. Previous study has demonstrated that CD177^+^ neutrophils play a protective role in the pathogenesis of inflammatory bowel diseases (IBD) through increased IL-22 production and bactericidal activity.^4^

Among the immune cells that contribute to the maintenance of intestinal homeostasis, intraepithelial lymphocytes (IELs) play a crucial role in preserving the mucosal barrier intact, patrolling the epithelial layer, and synergizing with rushing neutrophils to initiate the timely primary defense against exogenous pathogens.^5^ Especially, TCRγδ^+^ IELs are essential for limiting epithelial penetration of resident commensal microbiota through antimicrobial effectors (i.e., RegIIIγ), which are dependent on intestinal epithelial cell (IEC)-intrinsic MyD88 signaling following dextran sulfate sodium (DSS)-induced mucosal damage, indicating a critical role of proper TCRγδ^+^ IEL functions in maintaining host-microbiota homeostasis following acute mucosal inflammation.^6,7^

Optimal spatial interactions and cooperation between neutrophils and IELs are essential for maintaining gut mucosal immune homeostasis in response to pathobiont challenges. Loss of the delicate immunoregulation of neutrophils and IELs compromises the intestinal barrier integrity and drives a series of pathogenic disorders in the gut, such as neutropenic enterocolitis, infections, and IBD.^8,9^ Notably, accumulating activated CD8^+^ IELs have been found to be closely associated with the pathogenesis of celiac disease and correlated with gastrointestinal manifestations present in coronavirus disease 2019 (COVID-19) patients infected by severe acute respiratory syndrome coronavirus 2 (SARS-CoV-2).^10,11^ However, the underlying mechanisms whereby neutrophils regulate IELs and collectively preserve the intestinal homeostatic status are still not fully understood. Here, we reported an unanticipated mechanism by which neutrophils restrain intestinal mucosal inflammation through regulating IEL functions, mediated by microbial metabolite dimethyl fumarate (DMF), and provided novel insights into manipulating IEL activation by DMF as a potential therapeutic approach for patients suffering intestinal inflammatory disorders, e.g., inflammatory bowel disease (IBD).

## Materials and methods

### Experimental animals

*Cd177**^−/−^*** mice were purchased from the KOMP at the University of California (Davis, California, USA), bred, and maintained in the animal facility of Tongji University School of Medicine. C57BL/B6 wild type (WT) mice (6–8 weeks) used for the establishment of the DSS-induced colitis model were purchased from the Shanghai SLAC Laboratory Animal Co. Ltd (Shanghai, China), which were also bred with *Cd177**^−/−^*** mice to generated *Cd177*^+/+^ mice and used for microbiota analysis as WT littermates of *Cd177**^−/−^*** mice. For co-housing experiments, 3-week-old *Cd177**^−/−^*** mice and WT littermates were co-housed for at least 4 weeks (co-housing) or still housed separately.^12^ These mice were raised in an independent ventilation cage under specific pathogen-free conditions with a 12-hour light cycle and were fed autoclaved food and water. Male mice were used at 8–10 weeks of age with 20–25 g of body weight. All animal experiments in this study were reviewed and approved by the Institutional Review Board for Animal Research of the Shanghai Tenth People’s Hospital of Tongji University (SHDSYY-2018-3912).

### Dextran sulfate sodium (DSS)-induced colitis model in mice

DSS-induced colitis model in mice was established as described previously.^13^ Briefly, WT and *Cd177*^−/−^ mice were treated with 2% DSS in drinking water for 7 days, then replaced with sterile water for another 3 days. On day 10, all mice were sacrificed. The severity of colitis was scored daily by recording standard parameters including body weight, diarrhea, bloody stool, and survival rates. Colon tissues were removed, fixed in 10% paraformaldehyde, embedded in paraffin, sectioned, and stained with hematoxylin and eosin (H&E). Histological scores were calculated by combining the scores for each of six parameters, including the degree of inflammation in lamina propria (0-none, 1-mild, 2-moderate, 3-severe), goblet cell loss (0-none, 1-mild/moderate, 2-severe), disrupted crypts (0-normal, 1-hyperplastic, 2-disorganization, 3-crypt loss), presence of crypt abscesses (0-absent, 1-present), mucosal erosion (0-absent, 1-present) and submucosal ulceration spread to transmural involvement (0-none, 1-submucosal, 2-transmural), for a maximum score of 12. Three to four worst regions were selected, scored, and then averaged to determine the final score.

### Isolation of intestinal IELs and lamina propria mononuclear cells

As described previously, ^14^ the intestines were carefully cleaned from the mesentery and flushed of the fecal contents. Intestines were opened longitudinally, cut into 0.5–1.0 cm pieces, and then washed with cold phosphate-buffered saline (PBS) to remove the fecal contents. After the digestion by 1 mM EDTA in PBS at 37°C for 2 × 20 minutes, primary IELs were collected in the supernatants and further purified via density gradient centrifugation with 40% and 75% Percoll-Roswell Park Memorial Institute (RPMI) solution. Colon tissues were then digested by collagenase A (1 mg/mL; Sigma-Aldrich, St. Louis, Missouri, USA) at 37°C for 30 minutes. The single-cell suspension was collected and further purified via density gradient centrifugation with 40% and 75% Percoll-RPMI solution. Lamina propria mononuclear cells (LPMCs) were then collected from the interface and suspended in 10% fetal bovine serum (FBS)-RPMI medium.

### Flow cytometric analysis

For cell surface staining, IELs and LPMCs were obtained from *Cd177^–/^^–^* mice and WT littermates, first incubated with Fc Block (BD Biosciences), and then stained with fluorochrome-conjugated mAbs against TCRγ/δ, TCRβ, CD4, CD8a and CD8b.2, respectively, for 30 minutes at 4°C.^15^ Concomitantly, the Live/Dead Fixable Dead Cell stain kits (Invitrogen, Eugene, OR) was used to exclude dead cells. For intracellular cytokine staining, IELs were treated with phorbol 12-myristate 13-acetate (PMA, 50 ng/mL; Sigma-Aldrich) and ionomycin (750 ng/mL; Sigma-Aldrich) for 5 hours at 37°C, along with the stimulation of brefeldin A (3 μg/mL; eBioscience) for the last 3 hours. Subsequently, these cells were harvested and processed for surface staining, followed by fixation and permeabilization for 30 minutes at 4°C. After three washes, intracellular staining was performed with fluorochrome-conjugated anti-IFN-γ, anti-IL-17A, anti-TNF-α, and anti-IL-10 mAbs. All stained samples were analyzed on a BD FACS Canto II Flow Cytometer. All data were processed using FlowJo software (Version 10.0.7, Tree Star; Ashland, Oregon, USA).

### Depletion of neutrophils in vivo

To delete neutrophils, mice were injected intraperitoneally at a dose of 100 μg of anti-mouse Ly6G antibody (clone 1A8, Bio X Cell; West Lebanon, New Hampshire, USA) in PBS on days 0, 3, 6 and 9 before DSS administration. Rat IgG2a (clone 2A3, Bio X cell) was used as isotype control.^16^

### Bacterial infection of mice

*Citrobacter rodentium* (CR) strain DBS100 (ATCC51459; American Type Culture Collection, Rockefeller, Maryland, USA) was generously provided by Dr. Youcun Qian (The Key Laboratory of Stem Cell Biology, Institute of Health Sciences, Shanghai Institutes for Biological Sciences, Chinese Academy of Sciences, Shanghai, China). CR were prepared by gently shaking bacteria overnight at 37°C in Luria-Bertani broth. Bacterial cultures were progressively diluted and plated on MacConkey agar plates to obtain the optimal loads of CFUs administered. For infection experiments, mice were fast for 8 hours before oral inoculation with 2 × 10^9^ CFUs of CR in a total volume of 100 μl per mouse. Mortality was monitored daily throughout the infection. The changes in body weights were assessed at the beginning of infection and every 2 days after infection.^17^

### Antibiotics administration

To deplete gut bacteria of SPF mice, quadruple antibiotics, including ampicillin (1 g/L; Sigma-Aldrich), metronidazole (1 g/L; Sigma-Aldrich), neomycin (1 g/L; Sigma Aldrich), and vancomycin (0.5 g/L; Wako), were mixed in sterile drinking water for 4 weeks as described previously.^18^ Antibiotics cocktails were given to mice continuously until sacrificed during the experiments.

### DMF treatment

Mice were given DMF (100 mg/kg) by oral gavage daily in an emulsion of 0.6% methocel from 0 to 10 days of DSS treatment. An equivalent volume of 0.6% methocel vehicle was served as control.

### Single-cell RNA sequencing (scRNA-seq) analysis

Single-cell suspensions were isolated from the mouse colon epithelial layer through a positive selection of CD45^+^ immuno-magnetic beads and underwent cell viability detection. Samples with viabilities >85% were available for scRNA-seq analysis based on NovaSeq6000 platform, and sequencing libraries were generated with BD Rhapsody protocol adapted from the BD Resolve system as described previously.^19^ In brief, single cells were captured into >200,000 microwells with cell barcodes and unique molecular identifier barcodes before cell lysis, and mRNA capture via ligation of poly A. Cells with mitochondrial RNAs >40% and expression of fewer than 200 genes were discarded. PCA and tSNE analysis were used for the single-cell-to-cell relation description. Graphcluster and K-mean were utilized for cell clustering, and the Wilcox rank-sum test was used for marker gene analysis. Gene ontology (GO) analysis was annotated from NCBI, UniProt, and Gene Ontology. Fisher’s exact test was applied to identify the significant GO categories, and FDR was used to correct the *p* values.

### Statistical analysis

All of the data were expressed as mean ± SEM and analyzed using GraphPad Prism 8. Two-group comparisons were performed using Student’s *t-test*. Multiple sample comparisons were performed using the Mann–Whitney test. Two-group comparisons for data with different treatments were analyzed and were performed using two-way ANOVAs with Tukey’s multiple comparisons test. *p < .05, **p < .01, ***p < .001 and ****p < .0001 were considered statistically significant.

## Results

### Neutrophils modulate IEL properties during gut inflammation

**Figure 1.**
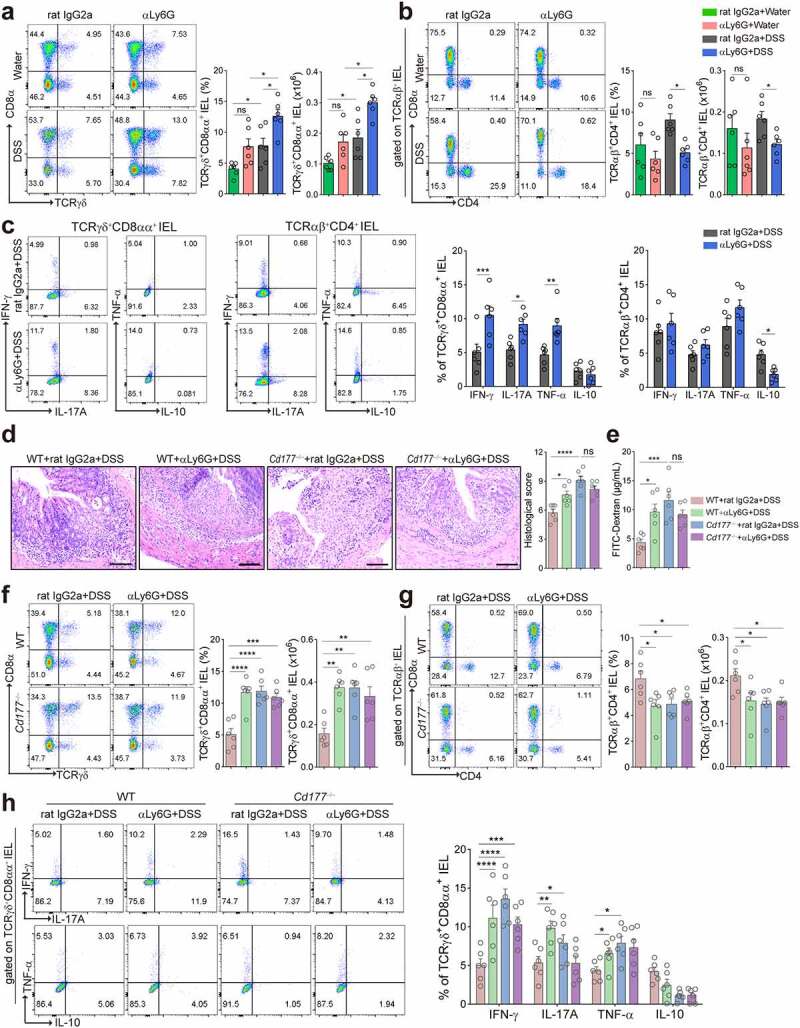
Elimination of neutrophils causes severe colitis and expansion of proinflammatory TCRγδ^+^CD8αα^+^ IELs in mice. Acute colitis was induced in WT mice (*n* = 6 per group) by 2% DSS in drinking water and treated intraperitoneally with anti-mouse Ly6G antibody (αLy6G) and rat IgG2a (100 μg/mouse), respectively, every three days. (**a, b**) Flow cytometric analysis of TCRγδ^+^CD8αα^+^ IELs (a) and TCRαβ^+^CD4^+^ IELs (b, gated TCRαβ^+^ IELs. The proportion of TCRαβ^+^CD4^+^ IELs is obtained by calculating the proportion of TCRαβ-CD4 double positive IELs) in the colon of the indicated groups on day 10. Bar charts showed the proportion and the absolute number of indicated IELs. (**c**) The frequencies of IFN-γ-, IL-17A-, TNF-α-, and IL-10-expressing TCRγδ^+^CD8αα^+^ IELs and TCRαβ^+^CD4^+^ IELs isolated from the colon of the indicated WT mice (*n* = 6 per group) were detected by flow cytometry and counted into the statistical chart. (**d-h**) Acute colitis was induced in WT and *Cd177^−/−^* mice (*n* = 6 per group) by 2% DSS in drinking water and treated intraperitoneally with αLy6G antibody and rat IgG2a (100 μg/mouse), respectively, every three days. (**d**) Representative H&E staining of the colon tissues from WT and *Cd177*^−/−^ mice treated with rat IgG2a or αLy6G antibody throughout the 10-day observation after DSS insults. Scale bar, 100 µm. Histological scores were shown as indicated (*n* = 6 per group). (**e**) Serum levels of FITC-dextran (4 kD, 600 mg/kg) in each group. (**f, g**) Flow cytometric analysis of TCRγδ^+^CD8αα^+^ IELs (f) and TCRαβ^+^CD4^+^ IELs (g, gated TCRαβ^+^IELs) in the colon of each group. Bar charts showed the proportion and the absolute number of indicated IELs. (**h**) The frequencies of IFN-γ-, IL-17A-, TNF-α-, and IL-10-expressing TCRγδ^+^CD8αα^+^ IELs isolated from the colon of colitic WT and *Cd177*^−/−^ mice treated with rat IgG2a or αLy6G antibody (*n* = 6 per group), assessed by flow cytometry and counted into the statistical chart. Data were representative of three independent experiments. *p < .05; **p < .01; ***p < .001; and ****p < .0001 and ns, no significant difference.

To investigate the role of neutrophils in modulating the IEL response, we first established an experimental model of acute colitis in WT mice induced by 2% DSS and concomitantly treated intraperitoneally with anti-Ly6G antibody to deplete neutrophils *in vivo* (Supplementary Figure S1a). Neutrophil-depleted mice developed more severe colitis than controls, characterized by significant weight loss, aggravated histological lesions, impaired mucosal barrier function, and expression of proinflammatory cytokines (e.g., IL-1β, IL-17A, IFN-γ and TNF-α) in the colon tissues (Supplementary Figure S1b-f). The depletion of neutrophils did not affect the compositions of IELs isolated from the colon at the steady-state (Figure 1a,b and Supplementary Figure S1g). However, we found that TCRγδ^+^CD8αα^+^ IELs increased in the colon of colitic mice, which were further upregulated in neutrophil-depleted colitic mice compared with controls (Figure 1a). TCRγδ^+^CD8αα^+^ IELs are largely exhausted during chronic inflammation, which are mainly considered a guard in the intestinal mucosa and contribute to pathogen restriction and tight regulation of innate and adaptive immune responses.^20^ TCRγδ^+^ cells expressing high gut-homing integrins (CD103 and α4β7) are considered ‘inflammatory’ cells due to the enhanced Th1/Th17 cell differentiation after the adoptive transfer.^21^ To investigate the properties of TCRγδ^+^CD8αα^+^ IELs during colitis, we examined the expression of different inflammatory cytokines by flow cytometry. Consistent with more severe colitis in neutrophil-depleted mice, higher proportions of IFN-γ-, IL-17A- and TNF-α-expressing TCRγδ^+^CD8αα^+^ IELs were detected in these mice compared to WT controls (Figure 1c). In contrast, TCRαβ^+^CD4^+·^IELs were found to be decreased in the colon of neutrophil-depleted mice during DSS-induced acute colitis (Figure 1b) and produced low levels of IL-10 (Figure 1c). Additionally, there were no consistent changes in the proportions and counts of TCRαβ^+^CD8αα^+^ and TCRαβ^+^CD8αβ^+^ IELs among these groups (Supplementary Figure S1g). Collectively, these data reveal that expanded TCRγδ^+^CD8αα^+^ IELs manifest with proinflammatory properties after neutrophil depletion, suggesting that neutrophils modulate IEL functions and maintain gut homeostasis during intestinal mucosal inflammation.

### TCRγδ^+^CD8αα^+^ IELs expand in colitic Cd177^−/−^ mice

Neutrophils have been shown to regulate the innate immune response at the early stage of inflammation.^22^ Our previous studies demonstrated that CD177^+^ neutrophils function as an activated subset of neutrophils with high bactericidal capacity, increased expression of IL-22 and TGF-β, and decreased release of proinflammatory mediators such as IFN-γ, IL-6, and IL-17A.^4^ Hence, we reasoned whether CD177^+^ neutrophils might predominantly modulate the expansion of TCRγδ^+^CD8αα^+^ IELs under inflammatory conditions. To this end, we induced acute colitis in *Cd177^−/−^* and WT mice with DSS insults and simultaneously depleted neutrophils with anti-Ly6G antibody intraperitoneally. We found aggravated mucosal damage and architectural distortion in the intestines of colitic *Cd177^−/−^* mice compared with WT controls, as manifested by aggravated histological lesions and damaged mucosal barrier (Figure 1d,e), indicating that *Cd177^−/−^* mice developed more severe colitis under DSS insults. Our previous studies have demonstrated no differences in immune cells in LPMC (such as CD4^+^, CD8^+^ T cells, and B cells) between *Cd177^−/−^* and WT mice under steady or inflammatory conditions,^4^ and we further observed no significant changes in the compositions of IELs isolated from the colon at the steady-state (Supplementary Figure S2a). Interestingly, we found that TCRγδ^+^CD8αα^+^ IELs were also increased, while TCRαβ^+^CD4^+·^IELs were decreased in the colon of the colitic *Cd177^−/−^* mice (Figure 1f,g). TCRγδ^+^CD8αα^+^ IELs in the colon of *Cd177^−/−^* mice also produced more IFN-γ,·IL-17A, and TNF-α than WT controls (Figure 1h). However, depletion of total neutrophils using anti-Ly6G antibody did not further affect the frequencies and cytokine production (e.g., IFN-γ,·IL-17A, and TNF-α) of TCRγδ^+^CD8αα^+^ IELs in colitic *Cd177^−/−^* mice compared with those in control IgG2a-treated *Cd177^−/−^* mice, indicating that CD177^+^ neutrophils mainly regulate TCRγδ^+^CD8αα^+^ IELs during colitis (Supplementary Figure S2b,c and Figure 1f).

As a complementary approach, we orally infected WT and *Cd177^−/−^* mice with *Citrobacter rodentium* (CR, 2 × 10^9^ CFUs/mouse), and found that *Cd177^−/−^* mice developed more severe colitis with more weight loss, impaired epithelial barrier integrity than WT controls after CR infection (Supplementary Figure S3a-c). Consistent with the results from DSS-induced colitis, exposure to CR infection also caused an increase in TCRγδ^+^CD8αα^+^ IELs but a decrease in TCRαβ^+^CD4^+·^IELs in the colon of *Cd177^−/−^* mice compared with WT controls (Supplementary Figure S3d,e). Taken together, these data demonstrated that CD177^+^ neutrophils control the colitogenic TCRγδ^+^CD8αα^+^ IEL expansion during colitis.

### TCRγδ^+^CD8αα^+^ IELs manifest with proinflammatory phenotype and hyper-responsiveness to microbiota

**Figure 2.**
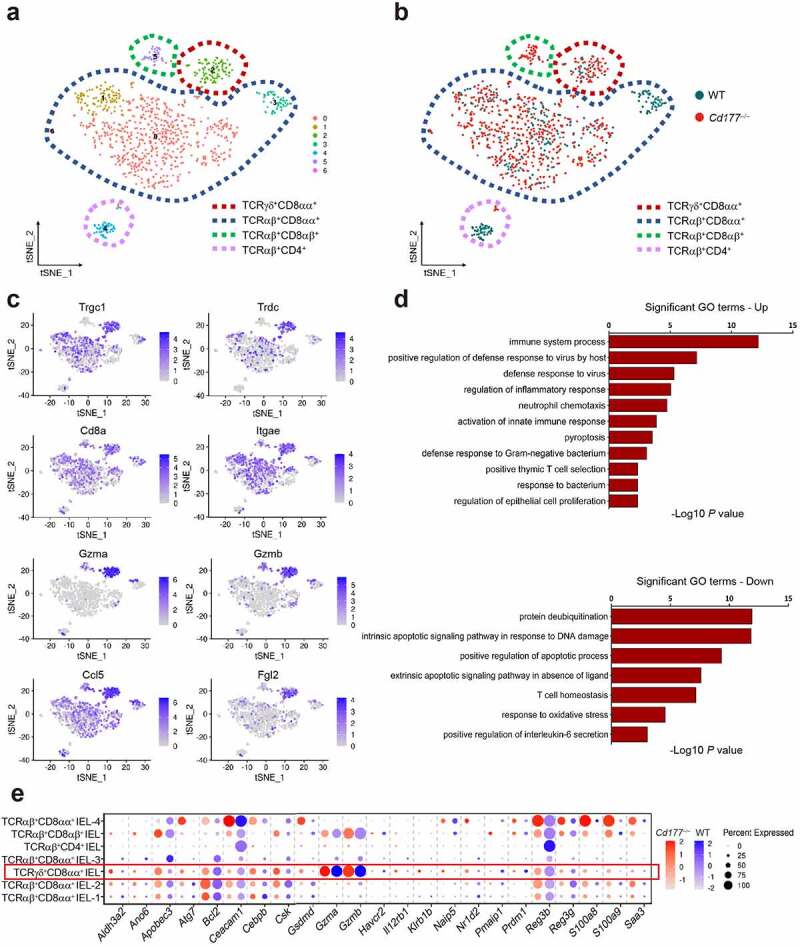
Gene expression signatures in TCRγδ^+^CD8αα^+^ IELs identify proinflammatory patterns and hyper-responsiveness to microbiota. A total of 14,301 single cells were isolated from mucosal epithelia of the colon of DSS-treated *Cd177**^−/−^*** mice and WT littermates through CD45^+^ immuno-magnetic bead sorting (*n* = 3 per group). (**a**) Unsupervised t-SNE analysis of IEL subclusters. (**b**) Unsupervised t-SNE analysis of IEL subclusters from DSS-treated *Cd177^−/−^* mice and WT littermates. (**c**) Identification of TCRγδ^+^CD8αα^+·^IELs (C2). (**d**) Gene Ontology (GO) analysis of upregulated and downregulated differentially expressed genes from TCRγδ^+^CD8αα^+·^IELs. (**e**) Dot plots of the selected gene expression in each cluster, colored by the average expression of each gene in each cluster, scaled across all clusters. The red and blue circles represent *Cd177*^−/−^ and WT mice, respectively. The depth of the color indicates the level of gene expression. Dot size represents the percentage of cells expressing the respective gene in each cluster.

To determine the functional properties of TCRγδ^+^CD8αα^+^ IELs during intestinal inflammation, we performed single-cell RNA sequencing (scRNA-seq) analysis of IELs isolated from DSS-treated colitic *Cd177**^−/−^*** mice and WT littermates to decipher the differentially expressed gene (DEG) profiles of IELs. A total of 14,301 single cells were isolated from colonic mucosal epithelia of DSS-treated colitic *Cd177**^−/−^*** mice and WT littermates, underwent CD45^+^ immuno-magnetic bead sorting, and were assigned to thirteen t-SNE unsupervised clusters (0–12) characterized by gene expression profiles (Supplementary Figure S4a). We conducted stepwise acquisition of IELs among all immune cell clusters, and cluster 7 was identified as IELs in terms of the enrichment of specific marker genes (e.g., *Cd3e, Ccl5, Gzma*, and *Gzmb*) (Supplementary Figure S4b). A focused clustering of IELs profiled seven clusters (C0-6) that were reassigned to four IEL categories (i.e., TCRγδ^+^CD8αα^+^, TCRαβ^+^CD8αα^+^, TCRαβ^+^CD8αβ^+^, and TCRαβ^+^CD4^+·^IELs) as assessed by flow cytometry previously (Figure 2a and Supplementary Figure S5a), and TCRγδ^+^CD8αα^+^ IELs were observed to be accumulated in *Cd177**^−/−^*** mice (Figure 2b). A focused re-analysis of IEL clusters revealed specific gene profiles, suggesting distinct features of different clusters. As for the large fraction of TCRαβ^+^CD8αα^+^ IELs in the colon (Supplementary Figure S1g and Figure S2b), they were further classified into four sub-clusters (i.e., C0, C1, C3, and C6) according to gene expression signatures (Supplementary Figure S5a,b). TCRαβ^+^CD8αα^+^ IEL-1 (C0) selectively expressed genes associated with intrinsic apoptotic signaling pathway in response to endoplasmic reticulum stress (e.g., *Cebpb*), intracellular signal transduction (e.g., *Irak2*), and regulation of transcription (e.g., *Tcf7, Ifnar1*). TCRαβ^+^CD8αα^+^ IEL-2 (C1) and the small cluster (TCRαβ^+^CD8αα^+^ IEL-4, C6) shared expression of genes involved in cell apoptosis and proliferation, such as *Mcm2, Mcm5, Cdx4, Lgals3*, and *Cldn7*. Interestingly, TCRαβ^+^CD8αα^+^ IEL-3 (C3) highly expressed *Nfkbid, Klf6*, and *Smad4*, suggesting an association with T cell receptor signaling and cytokine-mediated signaling.

Notably, we identified C2 as TCRγδ^+^CD8αα^+^ IELs by unique marker gene expression (e.g., *Trgc1, Trdc*, and *Cd8a*) (Figure 2c). The transcriptional signatures of TCRγδ^+^CD8αα^+^ IELs showed a significant enrichment of genes related to inflammatory response, activation of the innate immune response, neutrophil chemotaxis, and pyroptosis, accompanied by downregulation of genes involved in the modulation of T cell homeostasis, the response to oxidative stress and apoptotic process in colitic *Cd177^−/−^* mice, which was consistent with the aggravated mucosal inflammation and increased proportion of TCRγδ^+^CD8αα^+^ IELs in colitic *Cd177**^−/−^*** mice (Figure 2d). DEG analysis revealed that highly expressed genes encoding proinflammatory mediators (e.g., *Gzma, Gzmb, S100a8, S100a9, Fasl*, and *Klrb1b*) endowed TCRγδ^+^CD8αα^+^ IELs with profound cytotoxicity and antimicrobial properties (Figure 2e). Therefore, these data proved that neutrophil dysfunction triggers the functional alterations of TCRγδ^+^CD8αα^+^ IELs during gut inflammation, accompanied by disrupted intestinal mucosal homeostasis and microbial dysbiosis.

To assess whether the microbiota was associated with the changes in TCRγδ^+^CD8αα^+^ IELs when neutrophils were depleted, we analyzed scRNA-seq data related to microbial responses of TCRγδ^+^CD8αα^+^ IELs. We observed distinct gene expression profiles in TCRγδ^+^CD8αα^+^ IELs from colitic *Cd177^−/−^* mice, which were preferentially involved in bacterial invasion of the epithelial cells (74 genes including *Rhog, Arpc1a, Rac1, Pik3cb*, and *Arpc2*), defense response to Gram-negative bacteria (122 genes including *Lypd8, Gsdmd*, and *Naip5*), and response to bacteria (257 genes including *Mlh1, Acod1, Saa3, Slfn4, Saa1*, and *Trf*) (Figure 2d,e). These findings suggest that an alteration of microbial response may contribute to TCRγδ^+^CD8αα^+^ IEL activation and exacerbate mucosal inflammation in colitic *Cd177^−/−^* mice.

### Microbiota dysbiosis contributes to the expansion of TCRγδ^+^CD8αα^+^ IELs

**Figure 3.**
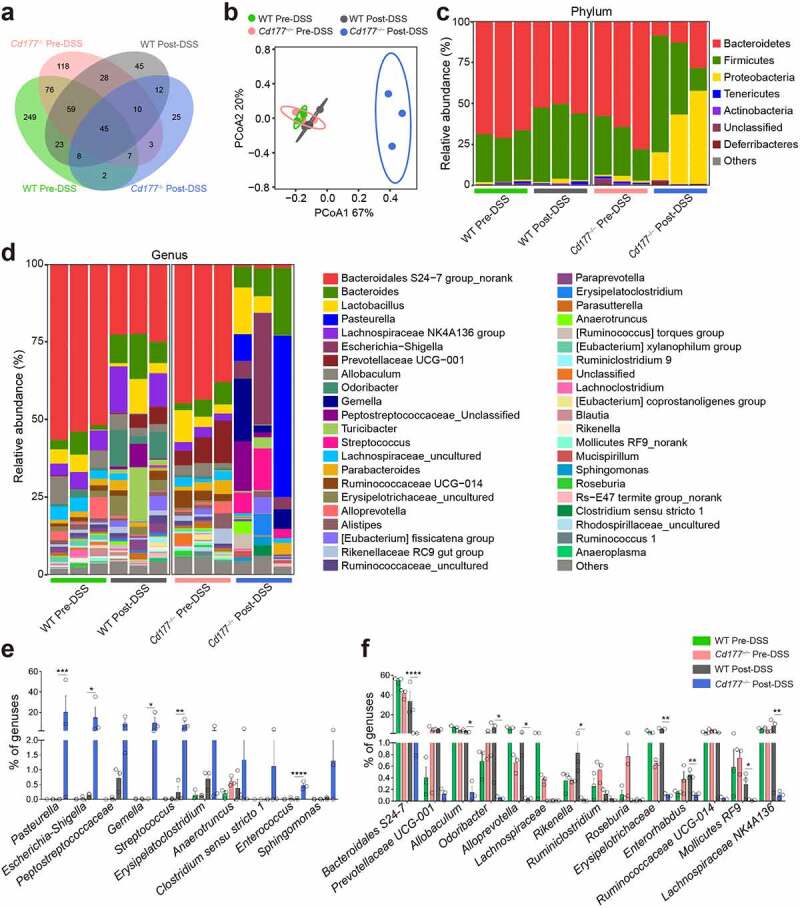
Microbial dysbiosis in DSS-induced colitic *Cd177*^−/−^ mice. (**a**) Differential OTUs of microbiotas in pre-DSS- and post-DSS-treated *Cd177*^−/−^ and WT mice. (**b**) PCoA plots of the bacterial communities (16S rRNA gene amplicons) in *Cd177*^−/−^ and WT mice feces pre- and post-DSS exposure (*n* = 3 per group). (**c, d**) Relative abundance of bacterial diversity of indicated groups at the phylum level (c) and the genus level (d). (**e**) Upregulated bacteria in the feces of *Cd177*^−/−^ mice after DSS exposure. (**f**) Downregulated bacteria in the feces of *Cd177*^−/−^ mice after DSS exposure. *p < .05; **p < .01; ***p < .001; and ****p < .0001.

Microbial dysbiosis is one of the significant consequences of neutrophil dysfunctions in numerous immune-mediated diseases such as IBD.^23^ We then investigated whether gut microbiota regulates TCRγδ^+^CD8αα^+^ IELs during colitis when neutrophils were depleted. We analyzed the microbial compositions in the feces of *Cd177^−/−^* and WT mice on days 0 and 10 during the period of DSS treatment. Figure 3a shows respective unique bacterial spectrums of colitic *Cd177^−/−^* mice and WT littermates captured by 16S rDNA gene amplicon sequencing. The beta diversity analysis was performed to compare the results of the principal co-ordinates analysis (PCoA) and revealed heterogeneity in colitic *Cd177^−/−^* mice compared to WT littermates (Figure 3b). Distinct bacterial ingredient ratios in both phylum and genus categories and a decreased diversity denoted a dysbiosis of the gut microbiota in colitic *Cd177^−/−^* mice (Figure 3c,d). Analysis of microbiome compositions further revealed a substantial decrease in protective bacteria (e.g., *Lachnospiraceae NK4A136 group, Rikenella*, and *Roseburia*),^24–27^ but the relative abundance in proinflammatory bacteria (e.g., *Escherichia−Shigella, Streptococcus, Enterococcus*, and *Peptostreptococcaceae_*Unclassified) in colitic *Cd177^−/−^* mice than in WT littermates (Figure 3e,f).^28–31^

To define the role of altered gut microbiota in neutrophil regulation of TCRγδ^+^CD8αα^+^ IELs, we conducted antibiotics treatment and co-housing procedure to eliminate the divergence in bacterial components between *Cd177^−/−^* mice and WT littermates (Supplementary Figure S6a). Treatment with an antibiotic cocktail (including ampicillin, metronidazole, neomycin, and vancomycin) in drinking water ameliorated colitis after DSS exposure, as evidenced by minor weight loss, decreased histological scores, mild impairment of mucosal barrier, and reduced expression of proinflammatory cytokines (e.g., IL-1β, IL-6, IL-17A, and IFN-γ) in the colon tissues of antibiotics-treated *Cd177^−/−^* mice compared with those in control *Cd177^−/−^* mice (Supplementary Figure S6b-f). There was no difference in the frequencies of TCRγδ^+^CD8αα^+^ IELs between antibiotics-treated *Cd177^−/−^* and WT mice after DSS insults (Supplementary Figure S7a). Moreover, reduced expression of proinflammatory cytokines (e.g., IL-17A, IFN-γ, and TNF-α) were found in TCRγδ^+^CD8αα^+^ IELs from antibiotics-treated colitic *Cd177^−/−^* mice (Supplementary Figure S7b). Consistently, *Cd177^−/−^* mice co-housed with WT littermates also showed alleviated mucosal inflammation after DSS exposure compared with singly housed *Cd177^−/−^* mice, as evidenced by mild weight loss and histological lesions, less impaired intestinal barrier, and reduced expression of proinflammatory cytokines (e.g., IL-1β, IL-6, IL-17A, and IFN-γ) in the colon tissues of co-housed *Cd177^−/−^* mice (Supplementary Figure S6b-f). Moreover, there were no differences in the frequencies of TCRγδ^+^CD8αα^+^ IELs between co-housed *Cd177^−/−^* mice and WT littermates (Supplementary Figure S7a). In line with antibiotics treatment, TCRγδ^+^CD8αα^+^ IELs from co-housed *Cd177^−/−^* mice expressed decreased levels of IL-17A, IFN-γ, and TNF-α (Supplementary Figure S7b). Collectively, these results indicate that microbial dysbiosis plays an important role in modulating TCRγδ^+^CD8αα^+^ IEL expansion and functions in *Cd177^−/−^* mice during colitis.

### Microbiota metabolite DMF decreases in colitic Cd177^−/−^ mice

**Figure 4.**
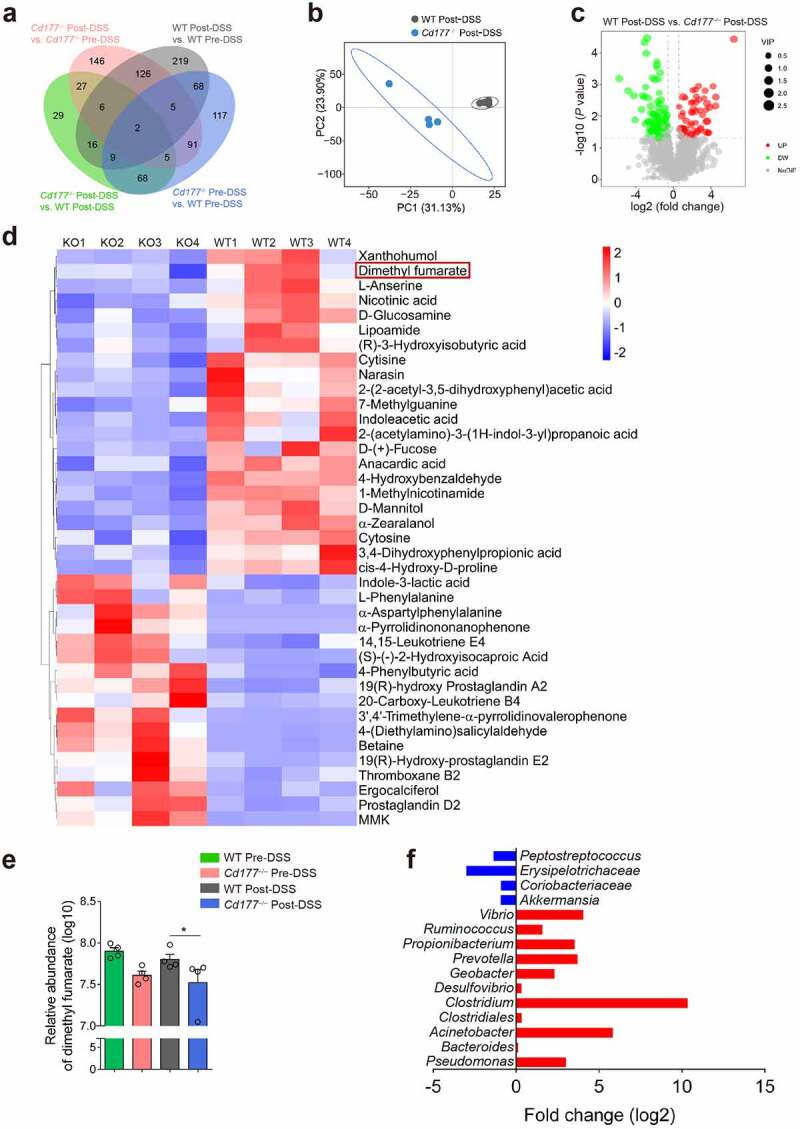
Alterations of the fecal metabolome in *Cd177*^−/−^ mice. Fecal samples from DSS-treated *Cd177*^−/−^ mice and WT littermates were collected on days 0 and 10 for unbiased metabolomics analysis (*n* = 4 per group). (**a**) Venn diagrams show the number of differently expressed metabolites detected in each group. (**b**) Partial Least Squares Discrimination Analysis (PLS-DA) of DSS-treated *Cd177*^−/−^ mice and WT littermates. (**c**) The volcano diagram represents all differentially expressed metabolites between DSS-treated *Cd177*^−/−^ mice and WT littermates. (**d**) Heatmap of differently expressed immunomodulatory metabolites between DSS-treated *Cd177*^−/−^ mice (KO) and WT littermates. (**e**) The levels of DMF in the fecal samples of the indicated groups. (**f**) Fold changes in fumarate-producing microbiota (blue) and fumarate-consuming microbiota (red) in the feces of colitic *Cd177*^−/−^ mice compared with colitic WT mice. *p < .05.

Given that microbiota metabolites profoundly affect the immunomodulatory effects of gut microbiota, we then performed an untargeted metabolomic analysis of the feces collected from *Cd177^−/−^* mice and WT littermates pre- and post-DSS insults to define the critical microbiota metabolites in regulating TCRγδ^+^CD8αα^+^ IELs. We found 29 overlapped metabolites in post-DSS-treated *Cd177^−/−^* and WT mice, 117 overlapped metabolites in pre-DSS-treated *Cd177^−/−^* and WT mice, and 219 overlapped metabolites in pre- and post-DSS-treated WT mice, and 146 overlapped metabolites in pre- and post-DSS-treated *Cd177^−/−^* mice (Figure 4a). These data were aligned better with the partial least squares discrimination analysis (PLS-DA), showing a significant heterogeneity of metabolome in colitic *Cd177^−/−^* mice (Figure 4b). To determine the specific metabolome signatures, differentially expressed metabolites were screened out (58 upregulated metabolites and 104 downregulated metabolites in colitic *Cd177**^−/−^*** mice) (Figure 4c). The functional analysis among these differentially expressed metabolites revealed that 17 upregulated metabolites (e.g., 14,15-leukotriene E4, thromboxane B2, prostaglandin D2, α-pyrrolidinononanophenone, and L-phenylalanine) were imprinted proinflammatory properties and that 22 downregulated metabolites (e.g., indoleacetic acid, xanthohumol, 3,4-dihydroxyphenylpropionic acid, α-zearalanol, 4-hydroxybenzaldehyde, anacardic acid, 1-methylnicotinamide, nicotinic acid, and cytisine) were related to immunoregulation (Figure 4d).

After determining the association between IEL changes and GSDMD-induced pyroptosis based on single-cell sequencing data, we then comprehensively analyzed the differential metabolites through microbial metabolomics data and screened out the differential expressed metabolites associated with regulation of pyroptosis in order to clarify the underlying mechanisms. Among these metabolites, DMF as a derivative of fumarate, a metabolic intermediate in the tricarboxylic acid (TCA) cycle converted from succinate by the enzyme succinate dehydrogenase, was markedly decreased in the fecal samples of colitic *Cd177**^−/−^*** mice (Figure 4d, e). DMF has shown broad anti-inflammatory effects on various immune cells, including neutrophils, dendritic cells, macrophages, NK, B, and T cells, and has already been used to treat patients with multiple sclerosis and psoriasis.^32^ Treatment with DMF decreases peripheral T cell counts, especially CD8^+^ T cells, in psoriasis patients through increased apoptosis and declined proliferation.^33^ Moreover, administration of DMF has been demonstrated to alleviate mucosal inflammation in the DNBS-induced murine colitis model.^34^ DMF-induced protein succination suppresses cleaved-gasdermin D (GSDMD)-mediated pyroptosis.^35^ Consistent with the decreased levels of DMF in the feces of colitic *Cd177**^−/−^*** mice, fumarate-producing bacteria (including *Akkermansia, Coriobacteriaceae, Erysipelotrichaceae*, and *Peptostreptococcus*) were decreased,^36,37^ while fumarate-consuming bacteria (including *Bacteroides, Clostridium, Acinetobacter, Vibrio, Propionibacterium, Prevotella, Clostridiales, Ruminococcus, Geobacter, Desulfovibrio* and *Pseudomonas*) were increased in colitic *Cd177**^−/−^*** mice (Figure 4f),^38–44^ suggesting that the altered gut microbiota was responsible for the decrease of DMF in the fecal samples of these *Cd177^−/−^* mice. Collectively, these data provide a hypothesis that microbial metabolite DMF may be associated with the regulation of IEL function and maintain intestinal mucosal homeostasis, and that deficiency of DMF may trigger intestinal mucosal inflammation and induce fundamental activation of pathogenic TCRγδ^+^CD8αα^+^ IELs.

### DMF supplementation restrains TCRγδ^+^CD8αα^+^ IEL pyroptosis and ameliorates intestinal mucosal inflammation

**Figure 5.**
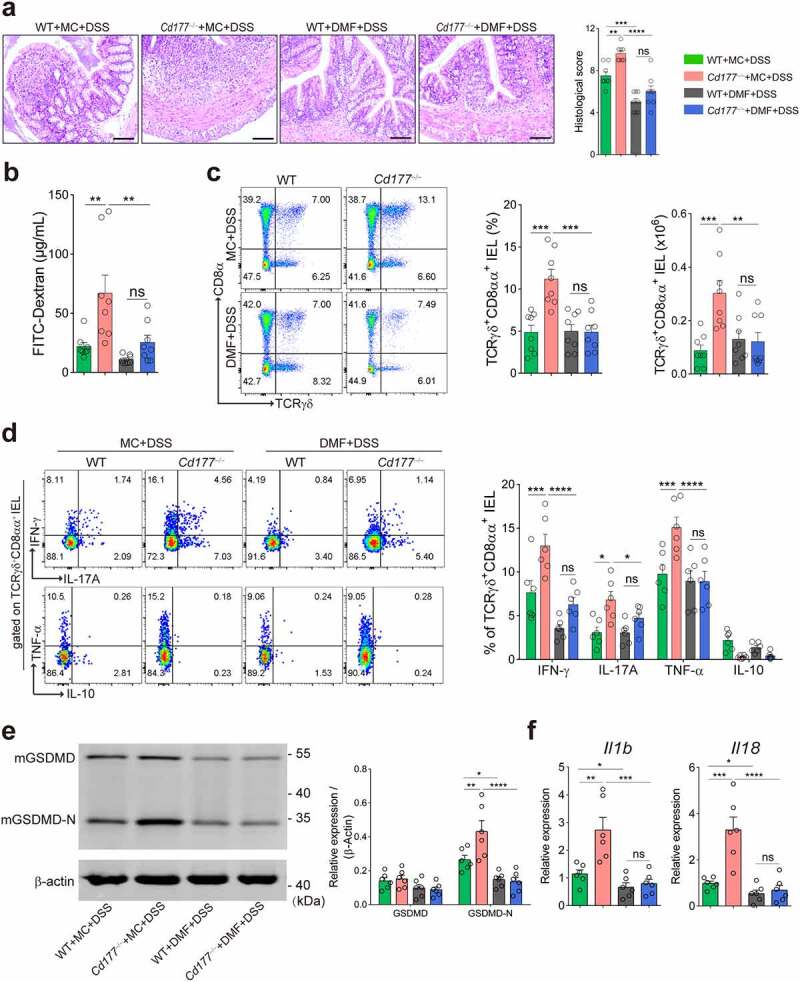
Administration of DMF ameliorates DSS-induced colitis in *Cd177*^−/−^ mice. Acute colitis was induced in WT (*n* = 8) and *Cd177*^−/−^ (*n* = 8) mice by 2% DSS in drinking water and treated orally with methocel (MC) or DMF (100 mg/kg) daily throughout the 10-day observation. (**a**) Representative H&E staining of the colon tissues was obtained from these mice on day 10. Scale bar, 100 µm. Histological scores were shown as indicated. (**b**) Serum levels of FITC-dextran in each group. (**c**) Flow cytometric analysis of TCRγδ^+^CD8αα^+^ IELs in the colon of each group on day 10. Bar charts showed the proportion and the absolute number of the indicated IELs. (**d**) The frequencies of IFN-γ-, IL-17A-, TNF-α-, and IL-10-expressing TCRγδ^+^CD8αα^+^ IELs isolated from the colon of colitic WT and *Cd177*^−/−^ mice treated with MC or DMF (*n* = 6 per group), assessed by flow cytometry and counted into the statistical chart. (**e**) The full-length GSDMD and GSDMD-N in flow-sorted TCRγδ^+^CD8αα^+^ IELs of indicated groups were determined by Western blot. Bar charts showed the relative expression of GSDMD and GSDMD-N. (**f**) The mRNA levels of pyroptosis-associated cytokines in flow-sorted TCRγδ^+^CD8αα^+^ IELs (*n* = 6 per group). Data were representative of three independent experiments. *p < .05; **p < .01; ***p < .001; and ****p < .0001 and ns, no significant difference.

We next sought to determine whether DMF supplementation inhibits the expansion of colitogenic TCRγδ^+^CD8αα^+^ IELs and intestinal inflammation. We treated *Cd177**^−/−^*** and WT mice with DMF by oral gavage and induced colitis with DSS (Supplementary Figure S8a). Administration of DMF substantially ameliorated colitis development in *Cd177**^−/−^*** mice, characterized by mild weight loss, lowered histological damage, and more intact epithelial barrier integrity (Supplementary Figure S8b and Figure 5a,b). Proinflammatory cytokines (e.g., IL-1β, IL-6, IL-17A, and TNF-α) were decreased in the inflamed colon of DMF-treated colitic *Cd177**^−/−^*** mice compared with the controls (Supplementary Figure S8c,d). Moreover, administration of DMF significantly inhibited TCRγδ^+^CD8αα^+^ IEL expansion in colitic *Cd177**^−/−^*** mice as well as the expression of proinflammatory cytokines in TCRγδ^+^CD8αα^+^ IELs (Figure 5c,d).

It has been shown that GSDMD expression is increased in inflamed mucosa of IBD patients by scRNA-seq analysis.^45^ Notably, we observed a significant increase in *Gsdmd* expression in TCRγδ^+^CD8αα^+^ IELs of colitic *Cd177**^−/−^*** mice compared with WT controls (Figure 2e). The pore-forming GSDMD-N was markedly increased in TCRγδ^+^CD8αα^+^ IELs of colitic *Cd177**^−/−^*** mice, as well as significantly higher expression of IL-1β and IL-18 (Figure 5e,f), suggesting that *Cd177**^−/−^*** TCRγδ^+^CD8αα^+^ IELs were more prone to pyroptotic cell death. However, DMF administration protected against TCRγδ^+^CD8αα^+^ IEL pyroptosis, as evidenced by reduced expression of GSDMD-N, IL-1β and IL-18 (Figure 5e,f). Consistent with these findings, higher levels of GSDMD-N were also detected in IECs and colon tissues of *Cd177**^−/−^*** mice after DSS insults but concomitantly blocked by DMF (Supplementary Figure S8f). Collectively, these data indicate that a decrease in microbial metabolite DMF due to the dysregulated immunoregulation of neutrophils on gut microbiota causes TCRγδ^+^CD8αα^+^ IEL expansion.

## Discussion

Intestinal resident IELs and dynamically circulating neutrophils are potent host defenders against gut pathogens due to the early response and robust cytotoxic effects. Their inappropriate functional status is associated with intestinal inflammatory diseases, such as IBD.^9,46^ Emerging evidence establishes the crucial role of neutrophils in the pathogenesis of IBD as a Janus-faced modulator relevant to aggravated intestinal inflammation and inflammation resolution gut mucosa in IBD, paradoxically.^46^ However, how neutrophils regulate IEL functions in the pathogenesis of IBD remains elusive, and the underlying mechanisms whereby neutrophils regulate the IELs in the context of a massive and diverse microbiota are not well defined. Our previous study demonstrated that CD177^+^ neutrophils play a protective role in IBD through increased bactericidal activity and IL-22 production.^4^ However, we found no significant difference in IL-22 levels from the colon between colitic *Cd177*^−/−^ mice and WT littermates, thus we speculated that IL-22 does not affect IEL activation and expansion. Our study uncovered the unknown mechanisms by which neutrophils regulate IEL function through modulating gut microbiota and their metabolite DMF, which controls the expansion of colitogenic TCRγδ^+^CD8αα^+^ IELs. TCRγδ^+^CD8αα^+^ IELs have proinflammatory functional heterogeneity with an overarching integrated feature of increased GSDMD-mediated pyroptosis during intestinal inflammation. With an increase of antimicrobial peptides (e.g., *s100a8, s100a9*, and *Reg3b*) and defense response-related genes (e.g., *Gzma, Gzmb, Klrb1b*, and *Fasl*), TCRγδ^+^CD8αα^+^ IELs appear protective bactericidal activity and immunoregulatory potential in an appropriate activated state, while the overactivation causes the compromised mucosal homeostasis and aggravated inflammatory response, leading to severe tissue damage in the gut mucosa.

Gut microbiota has been found to be associated with TCRγδ^+^·IEL activation and cytokine interactions and is crucial in shaping the IEL compartment.^47^ Our study demonstrated that microbial homeostasis under the control of neutrophils enables fine-tuning the delicate balance between the protective and pathogenic characters of TCRγδ^+^CD8αα^+^ IELs, thus providing novel insights into how neutrophils modulate IEL immune response through the alterations in microbial metabolites, particularly a decline of DMF. Consistently, supplementation of DMF unequivocally alleviated experimental colitis and restrained the overactivation of TCRγδ^+^CD8αα^+^ IELs in colitic *Cd177**^−/−^*** mice. Additionally, deletion of neutrophil leads to dysbiosis in gut microbiota, and an overgrowth of invasive microbiota may also cross the barrier directly into contact with the epithelial and immune cells and activate TCRγδ^+^CD8αα^+^ IELs. From a broader perspective, DMF can also prevent the pyroptosis of IECs and even the entire intestinal tissue, which is not limited exclusively to immunoregulatory effects on immune cells in the intestinal tissue.^34^ Given that the maintenance of intestinal homeostasis requires the synergy of multiple immune cells, the interactions between IECs and IELs may also play an important role in maintaining the immune homeostasis and inflammatory response in gut mucosa. Thus, these findings illustrate an important role of DMF in regulating intestinal inflammation and maintaining gut immune homeostasis. Currently, the FDA has approved DMF (e.g., Tecfidera) to treat multiple sclerosis and psoriasis, but DMF can also induce lymphopenia and restrict glycolysis in lymphocytes.^48^ Therefore, clinical trials with DMF may be warranted to assess its efficacy and side-effects in the treatment of IBD patients. However, DMF has not yet been marketed in China, and we are looking forward to the opportunity to conduct clinical trials of DMF in IBD in the future.

Given the loss of a rapid, effective response against exogenous pathogens by neutrophils, early defense against pathogens that invade the intestine requires the innate immune cells of the intestinal immunity to take over. In the complex and delicate intestinal immune network where mucus, immune cells, and symbiotic flora act together, there is a delineated way for the microbiota to regulate immune cells through metabolites. Based on the fact that there are many patients with neutropenia and a series of complications, such as fever, infection, diarrhea, and compromised mucosal homeostasis in the gastrointestinal tract, it is essential to better understand the immune response and self-regulation system in the absence of neutrophils. In patients with intestinal immunopathology, abdominal pain, diarrhea, and hematochezia seriously lessen the quality of life. Hence, it is considered to understand and improve intestinal immunity at the onset of neutropenia. IELs, as the first line of defense in the intestinal immune system, are also affected and altered during the process of neutrophil disability. We found that DMF derived from microbial metabolites could regulate IELs, thus providing a novel clue for maintaining intestinal immune homeostasis. Collectively, our study shed some light on seeking promising therapies for neutropenia in patients suffering from systematic autoimmune diseases (e.g., systemic lupus erythematosus), immune-related disorders in the gastrointestinal tract, or receiving an immunosuppressant (e.g., azathioprine). Therefore, such a strategy to restore gut microbiota-derived metabolites or restrict TCRγδ^+^CD8αα^+^ IEL overactivation will provide an avenue for potential advanced treatment.

## Supplementary Material

Supplemental Material

## Figures and Tables

**Table:** 

CR	Citrobacter rodentium
DEG	differentially expressed gene
DMF	dimethyl fumarate
DSS	dextran sulfate sodium
GSDMD	gasdermin D
H&E	hematoxylin and eosin
IBD	inflammatory bowel diseases
IEL	intraepithelial lymphocytes
LPMC	lamina propria mononuclear cells
scRNA-seq	single-cell RNA sequencing
WT	wild type

## Data Availability

All data supporting the findings of this study are included in the article and/or the supplementary materials. The original data sets are also available from the corresponding author upon request.
