# Supplementary material for: Dysregulation of CD177+ neutrophils on intraepithelial lymphocytes exacerbates gut inflammation via decreasing microbiota-derived DMF
Source: Gut Microbes. 2023 Feb 2;15(1):2172668. doi: 10.1080/19490976.2023.2172668 (PMC9897772; doi:10.1080/19490976.2023.2172668)
Supplement: Supplemental Material [file KGMI_A_2172668_SM5144.zip › Supplementary information (1).docx]

**Supplementary Figures**

**Supplementary Figure S1.** Neutrophil depletion causes severe colitis and changes in IEL in mice after DSS insults. (**a**) Schematic of DSS-induced colitis model in WT mice (*n* = 6 per group) treated intraperitoneally with anti-mouse Ly6G antibody (αLy6G) and rat IgG2a (100 μg/mouse), respectively. (**b**) Weight change of each group throughout the 10-day observation (*n* = 6 per group). (**c**) Representative H&E staining of the colon tissues from rat IgG2a- and αLy6G-treated WT mice with or without DSS exposure, with histological scores of the indicated groups (*n* = 6 per group). Scale bar, 100 µm. (**d**) Mice from the indicated groups were deprived of water for 8 hours and gavaged with fluorescein isothiocyanate (FITC)-dextran (4 kD, 600 mg/kg) 4 hours before sacrifice on day 10. Serum samples were then obtained to detect the levels of FITC-dextran by fluorescence microplate reader at the excitation maximum of 493 nm and an emission maximum of 517 nm. (**e**, **f**) The mRNA levels of indicated cytokines were analyzed by qRT-PCR in the colon tissues (**e**) and by ELISA in culture supernatants of the colon tissues (**f**) from the indicated groups (*n* = 6 per group). (**g**) The frequencies of TCRαβ^+^CD8αα^+^ and TCRαβ^+^CD8αβ^+^ IELs isolated from the colon of the indicated groups were detected by flow cytometry and counted into the statistical chart (including proportion and counts of indicated IELs, *n* = 6 per group). Data were representative of three independent experiments. **p* < .05; ***p* < .01; ****p* < .001; and *****p* < .0001 and ns, no significant difference.

**Supplementary Figure S2.** Flow cytometric analysis of IELs from colitic *Cd177^−/−^* and WT mice. (**a**) The frequencies of TCRγδ^+^CD8αα^+^, TCRαβ^+^CD8αα^+^, TCRαβ^+^CD8αβ^+^, and TCRαβ^+^CD4^+^ IELs isolated from the colon of *Cd177^−/−^* and WT mice at the steady-state were detected by flow cytometry and counted into the statistical chart (including proportion and counts of indicated IELs) (*n* = 6 per group). (**b**) The frequencies of neutrophils isolated from the colon of *Cd177^−/−^* and WT mice after DSS insults with or without anti-Ly6G antibody treatment (*n* = 6 per group). (**c**) The frequencies of TCRγδ^+^ IELs isolated from the colon of *Cd177^−/−^* and WT mice after DSS insults with or without anti-Ly6G antibody treatment (*n* = 6 per group). (**d**) The frequencies of TCRαβ^+^CD8αα^+^ and TCRαβ^+^CD8αβ^+^ IELs isolated from the colon of *Cd177^−/−^* and WT mice after DSS insults with or without anti-Ly6G antibody treatment (*n* = 6 per group). Data were representative of three independent experiments.

**Supplementary Figure S3.** *Cd177^−/−^* mice develop severe colitis and altered IELs after CR infection. WT and *Cd177*^-/-^ mice (*n* = 6 per group) were treated intraperitoneally with αLy6G and rat IgG2a (100 μg per mouse), respectively, and then infected orally with CR (2×10^9^/mouse). (**a**) Weight change of each group throughout the 10-day observation. (**b**) Representative H&E staining of the colon tissues from WT and *Cd177*^−/−^ mice treated with rat IgG2a or αLy6G antibody throughout the 10-day observation after CR infection. Scale bar, 100 µm. Histological scores were shown as indicated. (**c**) Serum levels of FITC-dextran in each group. (**d**, **e**) Flow cytometric analysis of TCRγδ^+^CD8αα^+^ IELs (**d**) and TCRαβ^+^CD4^+^ IELs (**e**, gated TCRαβ^+^IELs) in the colon of each group on day 10. Bar charts show the proportion and the absolute number of the indicated IELs. (**f**) The frequencies of TCRαβ^+^CD8αα^+^ and TCRαβ^+^CD8αβ^+^ IELs isolated from the colon of CR-infected groups were detected by flow cytometry and counted into the statistical chart (including proportion and counts of indicated IELs). Data were representative of three independent experiments. **p* < .05; ***p* < .01; ****p* < .001; and *****p* < .0001 and ns, no significant difference.

**Supplementary Figure S4.** The landscape of gene expression profiles of IELs by scRNA-seq. (**a**) Unsupervised t-SNE analysis of CD45^+^ immune cells. CD45^+^ immune cells were pooled from *Cd177*^−/−^ mice (*n* = 3) and WT littermates (*n* = 3) after DSS treatment (including 7497 cells in *Cd177*^−/−^ mice and 6804 cells in WT littermates). (**b**) Heatmap of top ten differentially expressed genes per cluster.

**Supplementary Figure S5.** Gene expression profiles of IEL subsets by scRNA-seq. (**a**) Marker genes of each sub-cluster of IELs as described in Fig. 2a. (**b**) Highly expressed gene profiles of identified IEL sub-clusters.

**Supplementary Figure S6.** ABX and co-housing treatment change DSS-induced colitis and IELs in *Cd177^−/−^* and WT littermates. For ABX treatment, *Cd177*^−/−^ mice and WT littermates were pretreated with or without an antibiotic cocktail (ABX, including ampicillin 1 g/L, metronidazole 1 g/L, neomycin 1 g/L, and vancomycin 0.5 g/L) in drinking water for 4 weeks. Acute colitis was then induced in these mice (*n* = 6 per group) by 2% DSS in drinking water, and ABX was given orally throughout the 10-day observation. For co-housing treatment, 3-week-old *Cd177*^−/−^ mice and WT littermates were co-housed (CH) for 4 weeks before DSS modeling (*n* = 6 per group). (**a**) Schematic of DSS-induced colitis model in *Cd177^−/−^* and WT littermates treated with ABX or co-housing procedure (*n* = 6 per group). (**b**) Weight change of each group throughout the 10-day observation. (**c**) Representative H&E staining of the colon tissues from colitic WT and *Cd177*^−/−^ mice induced by DSS together with or without ABX treatment or co-housing treatment throughout the 10-day observation. Scale bar, 100 µm. Histological scores were shown as indicated. (**d**) Serum levels of FITC-dextran (4 kD, 600 mg/kg) in each group. (**e**) The mRNA levels of indicated cytokines were analyzed by qRT-PCR in the colon tissues of each group. (**f**) The levels of indicated proinflammatory cytokines were analyzed by ELISA in culture supernatants of the colon tissues from each group. Data were representative of three independent experiments. **p* < .05; ***p* < .01; ****p* < .001; and *****p* < .0001 and ns, no significant difference.

**Supplementary Figure S7.** Microbial homogeneity abrogates the bias in TCRγδ^+^CD8αα^+^ IELs. (**a**) Flow cytometric analysis of TCRγδ^+^CD8αα^+^ IELs in the colon of DSS-induced colitis mice throughout the 10-day observation. Bar charts showed the proportion and the absolute number of the indicated IELs. (**b**) The frequencies of IFN-γ-, IL-17A-, TNF-α-, and IL-10-expressing TCRγδ^+^CD8αα^+^ IELs isolated from the colon of each group (*n* = 6 per group), assessed by flow cytometry and counted into the statistical chart. Data were representative of three independent experiments. **p* < .05; ***p* < .01; ****p* < .001; and *****p* < .0001 and ns, no significant difference.

**Supplementary Figure S8.** DMF treatment ameliorates DSS-induced colitis in *Cd177^−/−^* and WT mice. (**a**) Schematic of DSS-induced colitis model in *Cd177^−/−^* and WT littermates orally treated with DMF and MC, respectively (*n* = 8 per group). (**b**) Weight change of each group throughout the 10-day observation (*n* = 8 per group). (**c**, **d**) The indicated proinflammatory cytokines in the colon tissues were analyzed by qRT-PCR (**c**) and in the culture supernatants of the colon tissues by ELISA (**d**) from the indicated groups (*n* = 8 per group). (**e**) Flow cytometric analysis of TCRαβ^+^CD4^+^ IELs (gated TCRαβ^+^IELs) in the colon of each group (*n* = 8 per group). Bar charts showed the proportion and the absolute number of the indicated IELs. (**f**) The full-length GSDMD and GSDMD-N in IECs and the colon tissues of indicated groups were determined by Western blot (*n* = 6 per group). Bar charts showed the relative expression of GSDMD and GSDMD-N. Data were representative of three independent experiments. ***p* < .01; *****p* < .0001 and ns, no significant difference.

Table S1. Clinical characteristics of human subjects.

|  | Stool samples | | |
| --- | --- | --- | --- |
|  | HC | CD | UC |
| Number of subjects | 6 | 6 | 6 |
| Age, y (mean ± SD) | 26 ± 1.7 | 36.7 ± 11.6 | 53 ± 11.8 |
| Gender, n (%) |  |  |  |
| Male | 3 (50) | 5 (83.3) | 3 (50) |
| Female | 3 (50) | 1 (16.7) | 3 (50) |
| Disease duration, m (mean ± SD) | N/A | 38.8 ± 34.0 | 80.6 ± 40.1 |
| Disease location, n (%) |  |  |  |
| L1 (ileal) | N/A | 1 (16.7) | N/A |
| L2 (colonic) | N/A | 0 | N/A |
| L3 (ileocolonic) | N/A | 5 (83.3) | N/A |
| L4 (isolated upper GI disease) | N/A | 0 | N/A |
| E1 (rectum) | N/A | N/A | 0 |
| E2 (left hemicolon) | N/A | N/A | 2 (33.3) |
| E3 (extensive colon) | N/A | N/A | 4 (66.7) |
| Complications, n (%) |  |  |  |
| Intestinal stenosis | N/A | 2 (16.7) | 2 (33.3) |
| Enterobrosia | N/A | 0 | 0 |
| Perianal diseases | N/A | 3 (50) | 0 |
| Fistula | N/A | 2 (33.3) | 0 |
| Medications, n (%) |  |  |  |
| 5-aminosalicylate | N/A | 2 (33.3) | 5 (83.3) |
| Azathioprine | N/A | 0 | 0 |
| Glucocorticoids | N/A | 0 | 2 (33.3) |
| Infliximab | N/A | 4 (66.7) | 2 (33.3) |

Table S2. Clinical characteristics of human subjects.

|  | Colonic mucosa samples | |
| --- | --- | --- |
|  | HC | CD |
| Number of subjects | 30 | 30 |
| Age, y (mean ± SD) | 54.5 ± 13.5 | 37.2 ± 12.2 |
| Gender, n (%) |  |  |
| Male | 20 (66.7) | 26 (86.7) |
| Female | 10 (33.3) | 4 (13.3) |
| Disease duration, m (mean ± SD) | N/A | 69.4 ± 50.3 |
| Disease location, n (%) |  |  |
| L1 (ileal) | N/A | 10 (33.3) |
| L2 (colonic) | N/A | 2 (6.7) |
| L3 (ileocolonic) | N/A | 18 (60) |
| L4 (isolated upper GI disease) | N/A | 0 |
| Complications, n (%) |  |  |
| Intestinal stenosis | N/A | 25 (83.3) |
| Enterobrosia | N/A | 11 (36.7) |
| Perianal diseases | N/A | 9 (30) |
| Fistula | N/A | 20 (66.7) |
| Medications, n (%) |  |  |
| 5-aminosalicylate | N/A | 15 (50) |
| Azathioprine | N/A | 6 (20) |
| Glucocorticoids | N/A | 4 (13.3) |
| Infliximab | N/A | 9 (30) |
