# Supplementary figures and images for: Dysregulation of CD177+ neutrophils on intraepithelial lymphocytes exacerbates gut inflammation via decreasing microbiota-derived DMF
Source: Gut Microbes. 2023 Feb 2;15(1):2172668. doi: 10.1080/19490976.2023.2172668 (PMC9897772; doi:10.1080/19490976.2023.2172668)

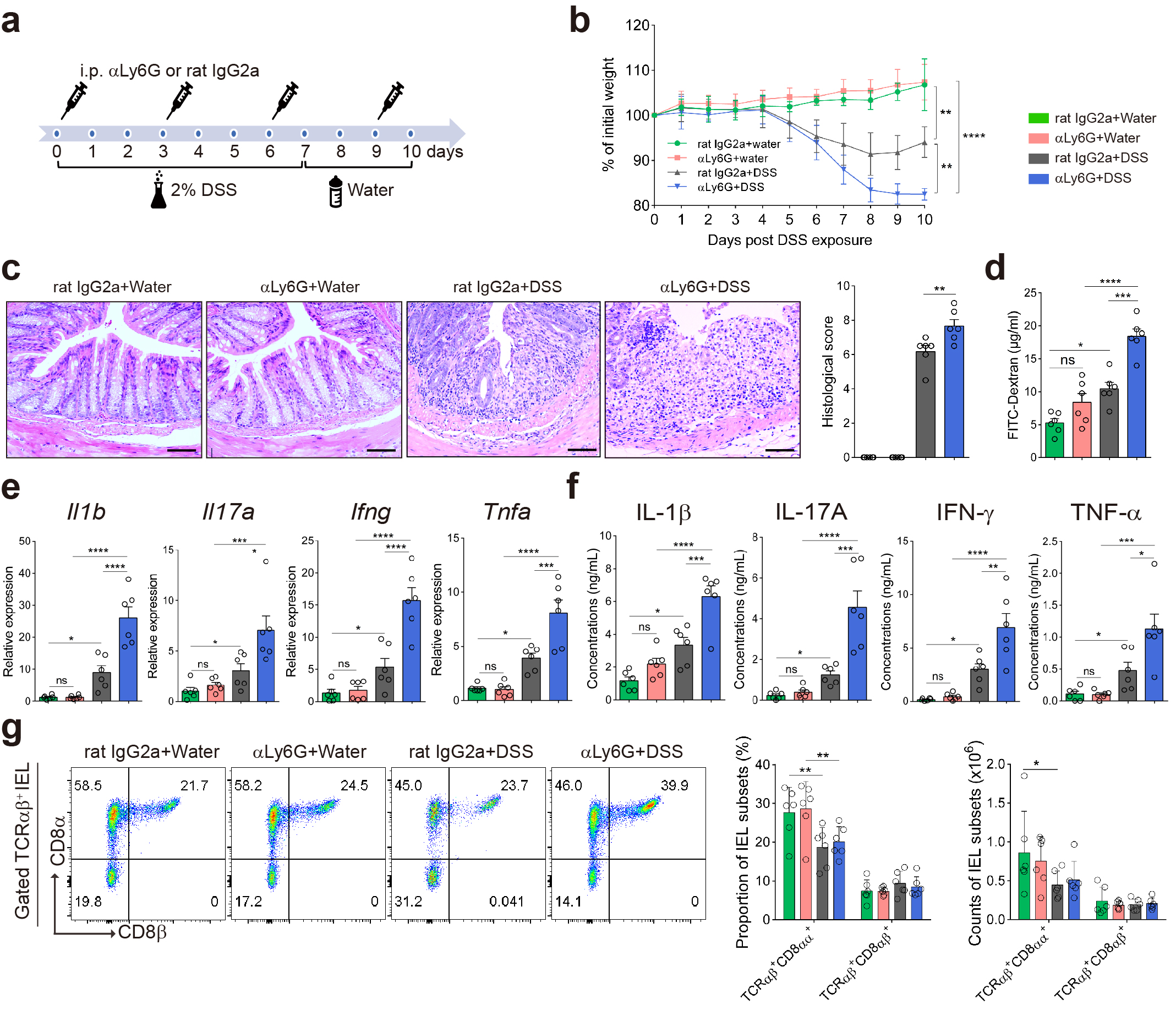

Supplement: Supplemental Material [file KGMI_A_2172668_SM5144.zip › Supplementary Figure 1.jpg]

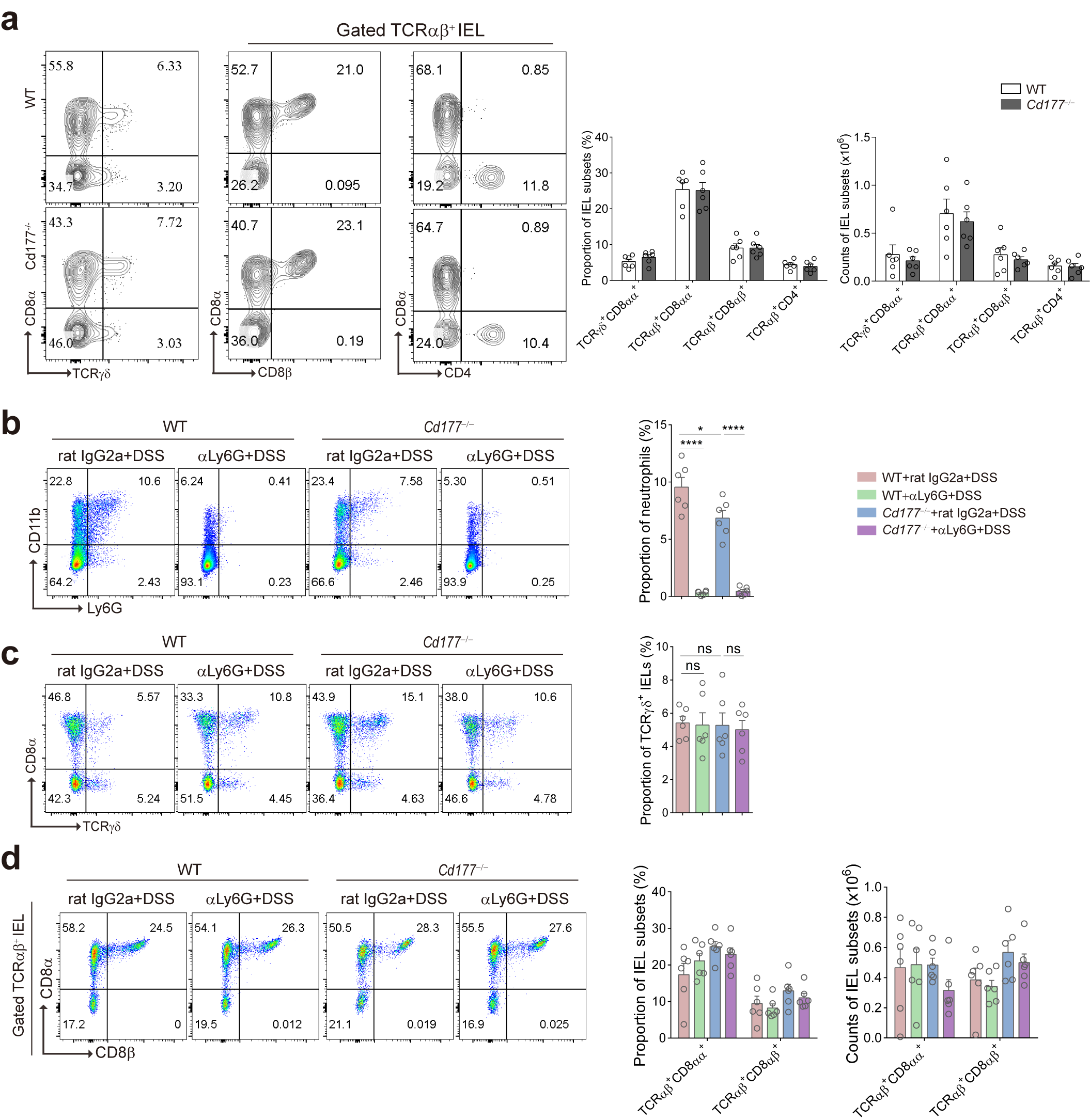

Supplement: Supplemental Material [file KGMI_A_2172668_SM5144.zip › Supplementary Figure 2.tif]

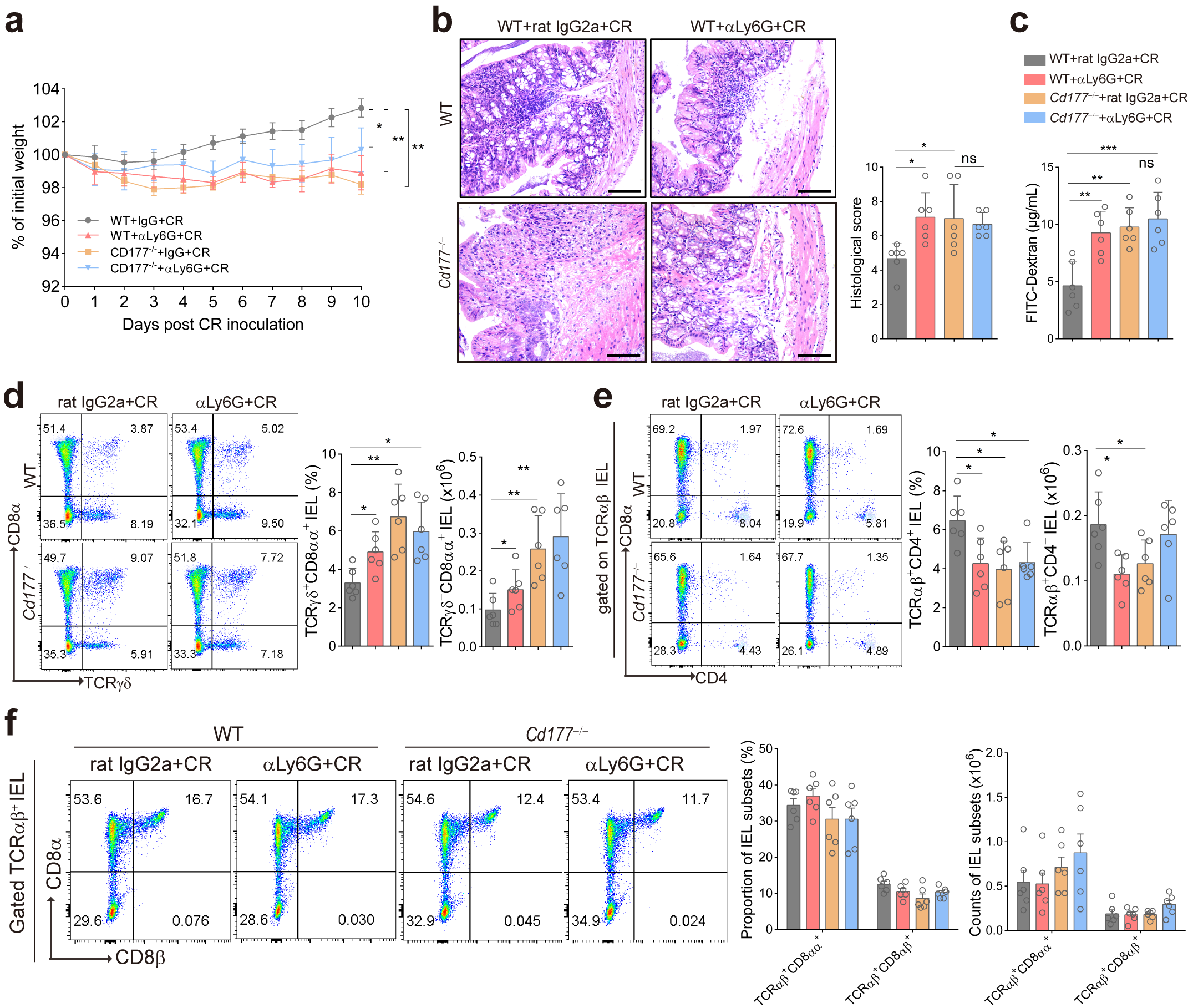

Supplement: Supplemental Material [file KGMI_A_2172668_SM5144.zip › Supplementary Figure 3.tif]

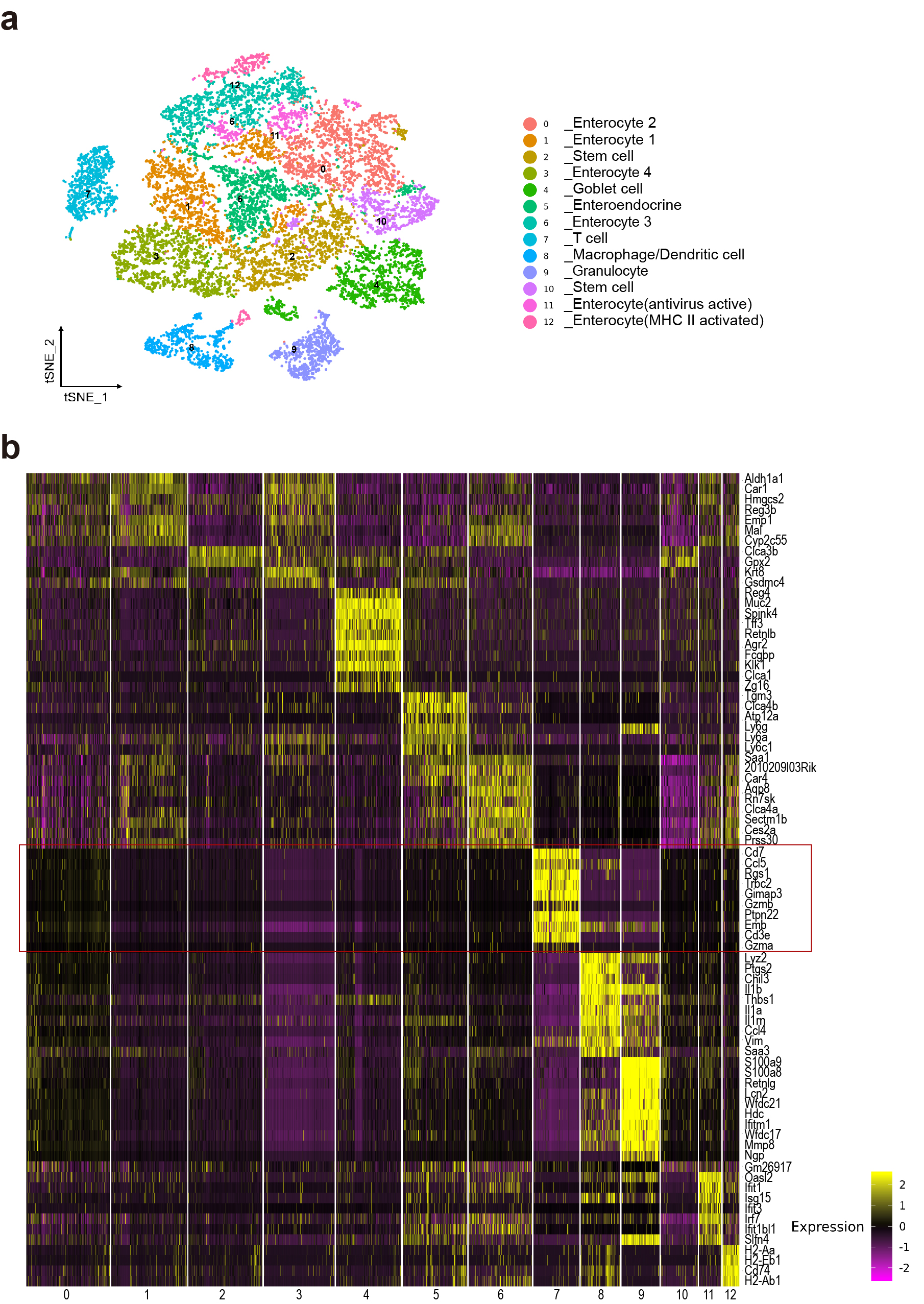

Supplement: Supplemental Material [file KGMI_A_2172668_SM5144.zip › Supplementary Figure 4.jpg]

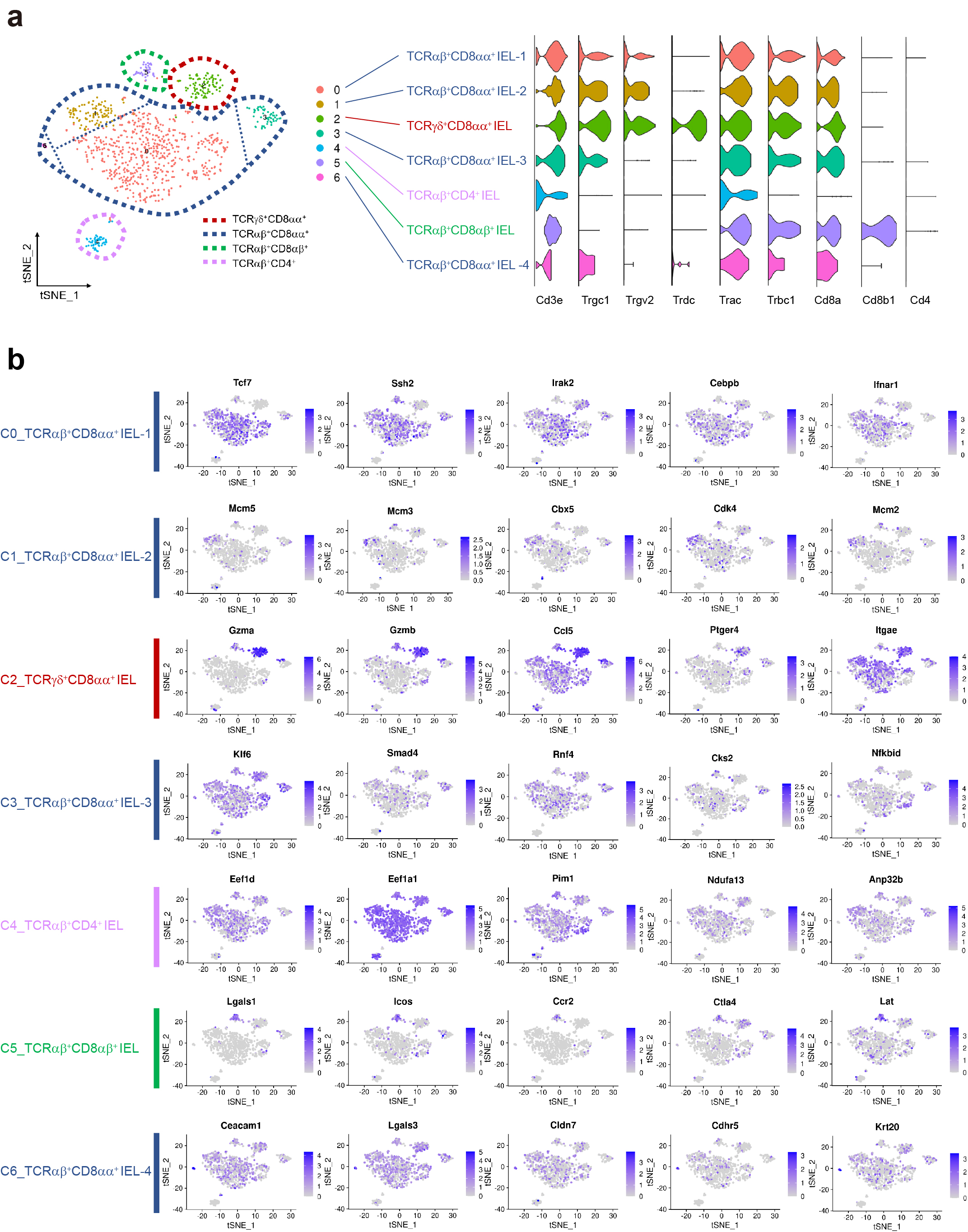

Supplement: Supplemental Material [file KGMI_A_2172668_SM5144.zip › Supplementary Figure 5.jpg]

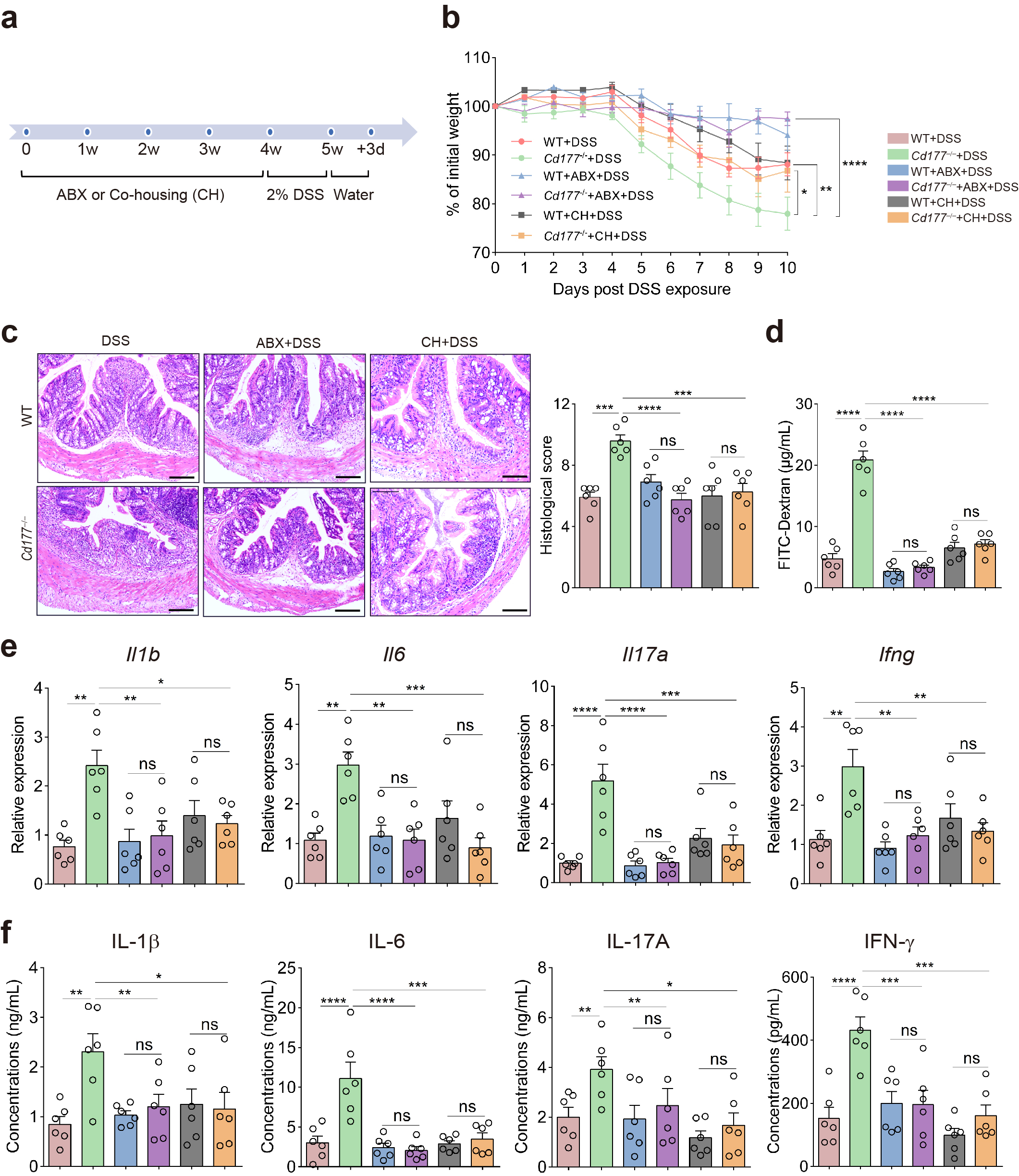

Supplement: Supplemental Material [file KGMI_A_2172668_SM5144.zip › Supplementary Figure 6.jpg]

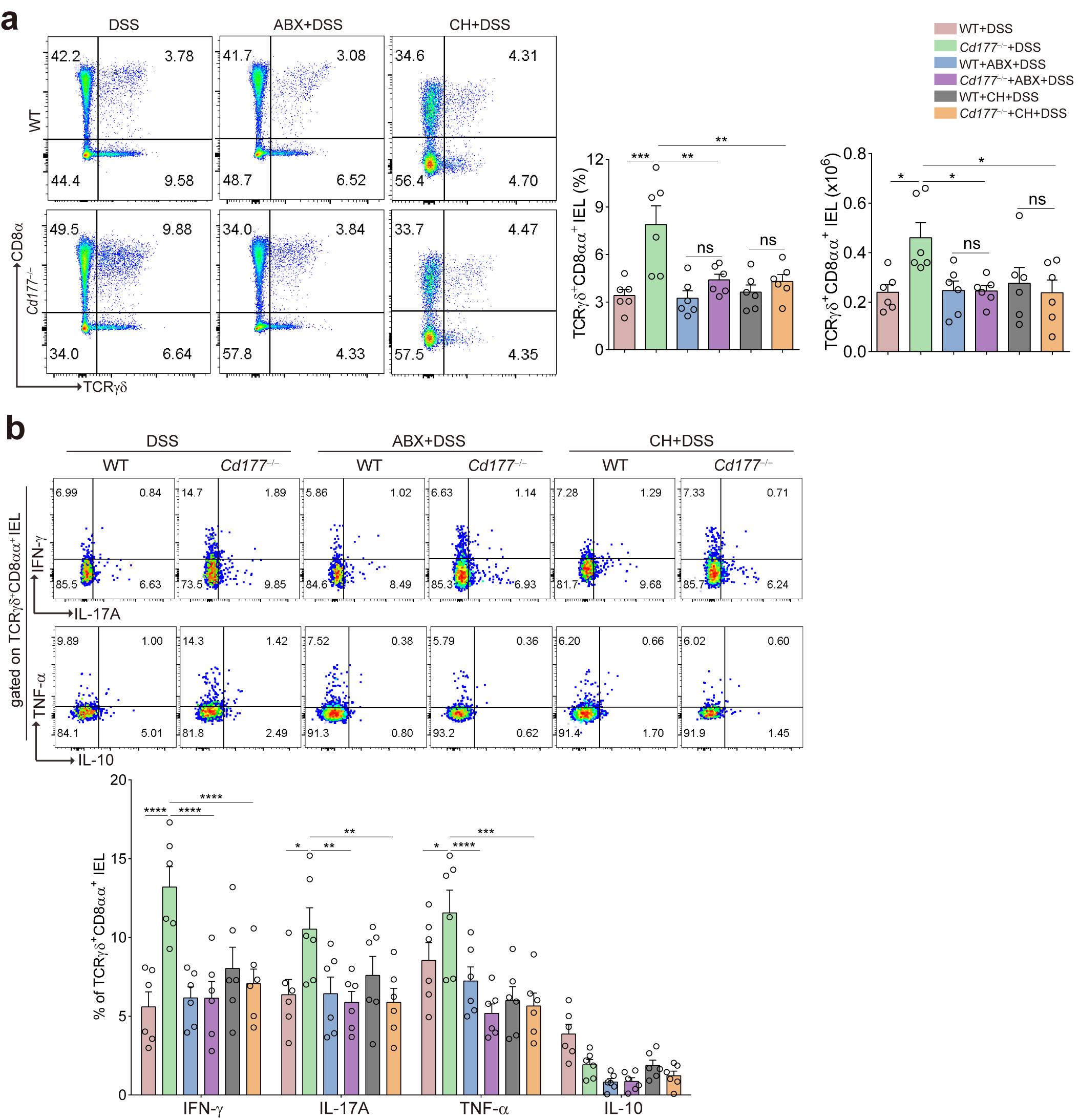

Supplement: Supplemental Material [file KGMI_A_2172668_SM5144.zip › Supplementary Figure 7.tif]

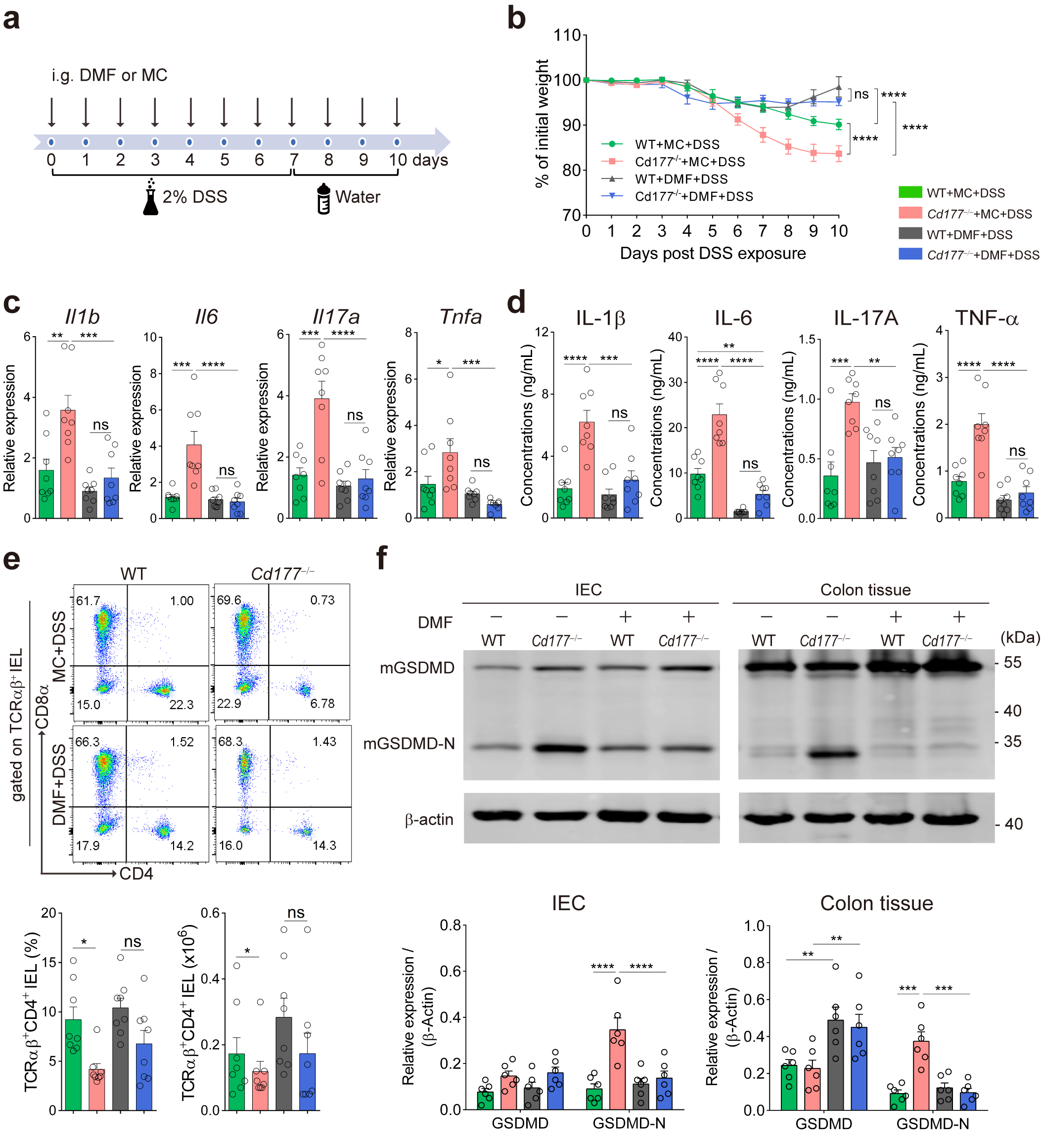

Supplement: Supplemental Material [file KGMI_A_2172668_SM5144.zip › Supplementary Figure 8.tif]
